# Effects of Mouthwash on Oral Cytomegalovirus, Epstein-Barr Virus, Herpes Simplex Virus Type-1

**DOI:** 10.52793/acmr.2022.3(4)-44

**Published:** 2022-11-08

**Authors:** Hessam Nowzari, Petra Wilder-Smith

**Affiliations:** 1 Private Practice, Beverly Hills, California, USA; 2 Professor, Director of Dentistry; Beckman Laser Institute, University of California, Irvine; 1002 Health Sciences Rd East, Irvine, CA 92617, USA

**Keywords:** Cytomegalovirus, HCMV, Periodontitis, CMV, Epstein-Barr virus

## Abstract

**Background:**

Cytomegalovirus (HCMV), Epstein-Barr virus (EBV), and herpes simplex virus type-1 (HSV-1) are pathogens.

**Objectives:**

The goal of the present double-blinded, randomized study was to compare the effect on oral viral load of twice daily use over 60 days of Lumineux Mouthwash^R^vs. de-ionized water. The main composition of the mouthwash was Dead Sea salt.

**Methods:**

30 participants were randomized to test or control. For 60 days, participants rinsed for 60s twice daily with 20ml of their allocated mouthwash, after morning and evening meals. On Day 0 and 60, before eating and oral hygiene and at least 60 minutes after drinking, unstimulated saliva was collected. Samples underwent mRNA analysis. Study endpoints were changes in Log Salivary Viral Load.

**Result:**

After adjusting for baseline differences, the reduction in viral load was significantly greater for the test group, all p-values <0.001. Baseline differences did not have an effect on the differences between groups in change over time.

**Conclusion:**

After adjusting for baseline differences, the reduction in viral load was significantly greater for the test group, all *p*-values <0.001. Baseline differences did not have an effect on the differences between groups in change over time.

## Introduction

Human cytomegalovirus (HCMV), Epstein-Barr virus (EBV), and herpes simplex virus type-1 (HSV-1) are emerging as major pathogens, in particular in immune compromised individuals [1]. Herpesviruses and oral bacteria may interact synergistically in causing significant infections which can be associated with severe clinical consequences.

Studies by Contreras et al. have presented strong evidence for the role of herpesviruses in the pathogenesis of human periodontal disease [3]. Contreras et al. determined the presence of herpes viruses in polymorphonuclear neutrophils, monocytes, macrophages and T and B lymphocytes in biopsies of periodontitis lesions from 20 adults. Periodontitis-derived monocytes and macrophages revealed HCMV in cell fractions from 11 (55%) patients and HSV in cells from 1 (5%) patient. T lymphocytes harbored HCMV in cell fractions from 4 (20%) patients and HSV in cell fractions from 4 (20%) patients. B lymphocytes showed EBV-1 in cell fractions from 9 (45%) patients. The study suggested that HCMV infects periodontal monocytes, macrophages and less frequently T lymphocytes and that EBV-1 infects periodontal B lymphocytes.

Our group studied active periodontitis lesions in 19 trisomy-21 patients detecting HCMV in 26% of patients [4]. In healthy periodontal sites, only one revealed HCMV. Subgingival debridement using a combination of hand and ultrasonic instruments did not reduce the presence of genomic herpesvirus. Viral-bacterial co-infections were observed in trisomy-21–associated destructive periodontal disease. We suggested that viral infection may reduce periodontal defense mechanisms and promote growth of putative periodontopathic bacteria such as *Tenarellaforsythensis*, *Prevotella intermedia*, and *Capnocytophaga* species. HCMV–Actinobacillusactinomycetemcomitans co-infection in localized aggressive periodontitis was reported by Nowzari et al. in a patient suffering from Fanconi anemia [5]. Nowzari et al. also analyzed HCMV pp67-mRNA amplification in oral fluids of 38 renal transplant recipients at 6 months post-transplantation [6]. Although patients had received antiviral therapy until 3 months post-transplantation, HCMV gene transcripts were detected in the saliva of 21% and the gingival crevicular fluid of 18% of patients. All patients (100%) with HCMV pp67-mRNA detected in saliva demonstrated clinical manifestations of viral infection, as did 86% of patients with HCMV pp67-Mrna detected in the gingival crevicular fluid. Transplant complications requiring urgent hospitalization were observed in 15 patients. Six of these patients were diagnosed with gingival overgrowth and active HCMV-associated periodontitis.

HCMV, EBV, and HSV-1each occurs in about 5% of healthy periodontal sites and 50% of severe periodontitis lesions [1–2]. In progressive periodontitis sites, herpesvirus copycounts can exceed bacterial cell counts [1–2]. In 2022, Nowzari et al. evaluated the composition of Dead Sea salt, its *in vitro* cytotoxicity, and reported on its efficacy against oral bacterial leukotoxins, oral endotoxins and oral glucan sucrose [7]. The most predominant elements detected in Dead Sea salt were the water of crystallization (H2O, water that is found in the crystalline framework of salt and which is not directly bonded), magnesium 0 chloride (MgCl2), potassium chloride (KCl), sodium chloride (NaCl), calcium chloride (CaCl2), bromide (Br -) and sulfates (SO4). While no cytotoxicity was detected, Dead Sea salt was highly effective against leukotoxin, endotoxin, and glucan sucrase enzyme. The authors suggested that rinsing with Dead Sea salt has the potential to contribute to the prevention of oral infections and recommended clinical research.

The goal of the present single center, double-blinded, randomized *in vivo* study was to compare the effect on oral viral load in 30 individuals of twice daily use over a period of 60 days of Lumineux Oral Essentials Clean and Fresh Mouthwash (Oral Essentials, Beverly Hills, CA 90210) vs. de-ionized water. The main composition of the tested mouthwash is Dead Sea salt.

## Material and Method

### Participants

30 individuals who met inclusion/exclusion criteria were recruited by mass e-mails and word of mouth on and around the University of California, Irvine campus. They provided written, informed consent under University of California, Irvine IRB-approved protocol # 2020-5719. Participants met the following inclusion and exclusion criteria:

### Inclusion criteria

- Male or female aged 25–35
- Gingival Index>2 [8]
- Able to provide written informed consent
- Able to attend study visits
- Available for follow up on the telephone
- Minimum of 20 teeth
- Measurable salivary viral load for HSV-1, HCMV and EBV at baseline.

### Exclusion criteria

- Use of antibacterial mouth rinse within 3 months or during study
- Systemic or topical oral antibiotic, antiviral, antifungal medications within 3 months or during study
- Any dental treatment within 1 month or during study
- History of significant adverse effects following use of oral hygiene products such as toothpastes and mouth rinses or allergy to personal care/consumer products or their ingredients.
- Presence of any condition, abnormality, or situation at baseline that in the opinion of the Principal Investigator may preclude the volunteer’s ability to comply with study requirements, including completion of the study or the quality of the data.

The study was performed in full compliance with University of California, Irvine IRB protocol 2020-5719, and all clinical procedures were conducted in accordance with the Helsinki Declaration of 1975, as updated in 2013. No changes were made in the study design after commencement of the study. Participants were randomized in a 1:1 ratio (randomizer.com) to use either the test rinse (Lumineux Oral Essentials Clean and Fresh Mouth wash, Oral Essentials, Beverly Hills, CA 90210), or a negative control rinse (de-ionized water (University of California, Irvine storehouse). Mouthwash bottles were masked to conceal the rinse’s identity from study participants and investigators. Subjects were asked to store and return all used mouth rinse containers to enable verification of usage compliance. Participants were contacted by telephone weekly to monitor and reinforce compliance. They were asked to keep up any pre-existing hand washing and mask-wearing routine, not to change other hygienic habits, and not to take any cold remedies during the intervention period. Participants maintained a daily health log, recording presence, duration, as well as any signs or symptoms that deviated from full health. This log included any un-wellness, including any upper respiratory tract infections (URTI) complaints such as nasal symptoms (rhinorrhea and sneezing), pharyngeal symptoms (soreness and scratchiness), bronchial symptoms (cough and phlegm), and general symptoms (feverishness, arthralgia, malaise, and any other deviations from full health). Each symptom was classified into four grades, that is, none (0), mild (1), moderate (2), and severe (3), according to the Jackson method. “Mild” was defined as when a participant was unaware of the symptom when he/she was busy; “moderate” as when one always felt discomfort; and “severe” as when one experienced difficulty in activities of daily life.

For 60 days, after shaking the bottle thoroughly, participants rinsed for 60s twice daily with 20ml of their allocated mouthwash, directly after morning and evening meals. They abstained from food and drink for at least 30 mins after rinsing. On Day 0, before eating and oral hygiene, before mouthwash use had begun, and at least 60 minutes after drinking, unstimulated saliva was collected. Participants were asked to accumulate saliva in the floor of the mouth and spit it out into a graduated Zymo Collection Tube^R^ every 60 seconds for 5 minutes, then to shake it vigorously to ensure proper stabilization. Saliva was again collected in the same way on Day 60 of the study. Samples were frozen in an −800°C freezer, where they were stored until all the samples were acquired and were processed together. Saliva samples underwent mRNA analysis using RT-PCR of viral load of HSV1, CMV, and EBV (Thermo-Fischer Scientific, Waltham, Mass 02451, USA). Study endpoints included (a) changes in Log Salivary Viral Load (HS-1, HCMV and EBV) Day 60 vs Day 0, and (b) presence and severity of any illness and of URTI-specific symptoms on the health log.

## Results

### Participants

All participants completed the study in full compliance with the protocol. Their demographics are shown in (Table 1). No adverse events were reported or observed.

### Viral Load

#### Baseline viral load

The Test group baseline means were significantly higher compared to the Control group for CMV and EBV (Two Group T-test. For HSV-1, the Test group baseline mean was lower than for the Control group. The difference approaches significance (Two Group T-test) (Table 2–4).

The reduction in viral load (change Day 0 – Day 60) was significantly greater for the Test group than for the Control group for all 3 viruses. The first analysis shown in (Table 5) ignores any possible influence of differences due to baseline values and merely analyzes change over time. The reduction in viral load was found to be significant for all 3 viruses (p<0.1), and highly significant for CMV and EBV (p≤0.05) (Two-Group t-Test for Difference in Paired Change Values). After adjusting for baseline differences (Table 6), the significance of differences between groups in change over time increased, with all *p*-values <0.001. Adjusted values for mean differences were slightly smaller than the unadjusted differences for HSV and CMV, but larger for EBV. Baseline differences did not have an effect on the differences between groups in change over time. These differences remain significant after adjusting for baseline values (Repeated Measures ANOVA Adjusting for Baseline (Day-0) Value).

In the Control group, participants recorded 5 health events: (1) Moderate URTI week 2; (2) Moderate cough week 4; (3) COVID-19 week 5; (4) COVID-19 week 6; (5) COVID-19 week 7. In the Test group, 2 health events were recorded: (1) Moderate food poisoning week 4; (2) COVID-19 week 6. There was no significant difference in frequency of health log entries between the 2 groups (p=0.195) (Chi-square test).

## Discussion

In 2013, Michel, et al. in a study entitled “The street children of Manila are affected by early-in-life periodontal infection: description of a treatment modality: sea salt” examined the effect of Sea Salt in 617 abandoned children who were living in the streets of Manila in the Philippines and provided evidence of the effectiveness of sea salt in the reduction or elimination of periodontal bacterial pathogens [9]. In 2017, Rodriguez and Ajdaharian evaluated the effects of the same mouthwash used in the present study to improve gingival health in an *in vivo* prospective, randomized, controlled, double-blinded study and reported significant reduction in gingival inflammation [10].

The tested mouthwash contains the elements of sodium and chlorine, iodine, magnesium, sulfur, calcium, potassium, phosphorus, fluorine, titanium, beryllium, germanium, and zinc [7–11] Sukenik, et al. evaluated the efficacy of Dead Sea balneotherapy in patients suffering from osteoarthritis of the knees in a randomized controlled study and provided evidence of significant improvement as measured by the Lequesne index of severity of osteoarthritis [12]. The improvement lasted up to 3 months of follow-up. Katz, et al. in a systematic review assessed the level of evidence for the claims of therapeutic effects of Dead Sea treatments in several rheumatologic diseases and psoriasis as well as reviewed these treatments’ safety [13,14]. Dead Sea salt was found to be beneficial in several rheumatologic diseases and psoriasis with a good safety profile.

In the present study, the Test group baseline means were higher compared to Control group for HCMV and EBV. For HSV-1, Test group baseline mean was lower than for Controls. However, baseline differences did not have an effect on the differences between groups in change over time. Paired differences (change Day 0 – Day 60) were significantly greater for the Test group than for the Control group for all 3 viruses. After adjusting for baseline differences (Table 5,6), the significance of differences between groups in change over time increased even further, with all *p*-values <0.001. Adjusted values for mean differences were slightly smaller than the unadjusted differences for HSV and HCMV, but larger for EBV.

Personal health-care plays an important role in lowering health-care cost. A simplified preventive approach that can reduce or eliminate herpes viruses could eliminate the need for expensive and complex treatments [1,2,4,6]. Herpes virus species are the most prevalent viruses in human saliva [1]. Eight herpes virus species can infect humans: herpes simplex virus-1 and -2, varicella-zoster virus, Epstein–Barr virus, human cytomegalovirus, human herpesvirus-6, human herpesvirus-7 and human herpesvirus-8 (Kaposis sarcoma virus) [1]. Herpes viruses establish a lifelong persistent infection, and some herpes virus species infect as many as 90% of the adult population.^14^ The clinical outcome of a herpes virus infection ranges from subclinical or mild disease to encephalitis, pneumonia, mononucleosis, and various types of cancer [1,2].

In conclusion, daily rinsing with Oral Essential mouthwash was more effective in reducing viral loads of HCVM, EBV, and HSV-1 than water. Mouthwash rinsing presented high efficacy and tolerability. The key to reducing viral loads was easy-to-use, effective, and a safe preventive intervention.

## Figures and Tables

**Table 1:** Demographics of Study Participants. M-Male; F-Female; N-Not identifying as M or F; A-Asian; W-White; MR-Mixed Race; PI-Pacific Islander; H-Hispanic.

	Gender (M/F/N)	Race	Ethnicity	Age Range	Mean Age	Median Age
**Control Group**	9M; 7F; 0N	8A; 6W; 2MR	4H	25–34 y	28.0 y	28 y
**Test Group**	8M; 6F; 0N	9A; 4W;1PI	3H	25–35 y	29.8 y	29.8 y
**Total**	17M; 13F; 0N	17A;10W;2MR; 1PI	7H	25–35 y	28.9 y	28 y

**Table 2:** Descriptive Data, Test Group (OE), HSV1, CMV, EBV at Day 0, Day 60, and Difference (Day-0 - Day-60).

	HSV1 Day 0	HSV1 Day 60	HSV1 DIF	CMV Day 0	CMV Day 60	CMV DIF	EBV Day 0	EBV Day 60	EBV DIF
**N**	15	15	15	15	15	15	15	15	15
**Minimum**	2.16	1.84	·1.28	2.73	1.89	−0.37	4.07	1.68	·2.2
**Maximum**	7.63	4.51	−3.12	8.14	7.12	·2.42	10.42	5.82	·5.27
**Median**	4.49	2.99	·1.53	5.21	3.84	·1.37	8.86	4.38	−4.03
**MEAN**	4.469	3.022	−1.447	5.294	3.933	−1.361	8.011	4.085	−3.926
**S.E.**	0.404	0.207	0.271	0.433	0.394	0.15	0.526	0.33	0.237
**S.D.**	1.563	0.802	1.051	1.677	1.527	0.583	2.037	1.279	0.92

**Table 3:** Descriptive Data, Control Group (Water), HSV1, CMV, EBV at Day 0, Day 60, and Difference (Day-0 - Day-60).

	HSV1 Day 0	HSV1 Day 60	HSV1 DIF	CMV Day o	CMV Day 60	CMV DIF	EBV Day o	EBV Day 60	EBV DIF
**N**	15	15	15	15	15	15	15	15	15
**Minimum**	2.64	0.52	−2.01	4.1	0.31	·3.79	3.12	0.58	·2..3
**Maximum**	9.83	2.05	−7.82	10.33	1.41	−8.92	8.15	1.42	−6.73
**Median**	5.49	0.98	−4.21	7.69	0.9	0.6	6.09	1.15	−4.94
**MEAN**	5.62	1.146	−4.474	7.379	0.867	−6.512	5.958	1.009	−4.949
**S.E.**	0.483	0.144	0.367	0.482	0.087	0.409	0.43	0.076	0.368
**S.D.**	1.872	0.559	1.422	1.865	0.337	1.586	1.666	0.294	1.425

**Table 4:** Comparison of Baseline Salivary Load in Control and Test Groups.

	Control			Test								
	N	Mean	SD	N	Mean	SD	Mean Difference	Lower Cl	Upper Cl	t	df	p-Value
**HSV1 Day 0**	15	4.469	1.563	15	5.62	1.872	−1.151	−2.441	0.139	−1.828	28	0.078
**CMV Day 0**	15	5.294	1.677	15	7.379	1.865	−2.085	−3.411	−0.758	−3.219	28	0.003
**EBV Day 0**	15	8.011	2.037	15	5.958	1.666	−2.053	0.661	3.445	3.022	28	0.005

**Table 5:** Comparison of Change in Salivary Viral Load from Baseline to Day 60 between Control and Test AFTER Adjusting for Baseline Value

	Control			Test								
	N	Mean Change	SD	N	Mean Chanae	SD	Mean Difference	Lower Cl	Upper Cl	t	df	p-Value
		(Day 0-Day 60)			(Day 0-Day 60)		(CO chg-TST chg)					
**HSV1 DIF**	15	1.447	1.051	15	4.474	1.422	−3.027	−3.963	−2.092	−6.629	28	<0.001
**CMV DIF**	15	1.361	0.583	15	6.512	1.586	−5.151	−6.045	−4.258	−11.809	28	<0.001
**EBV DIF**	15	3.926	0.92	15	4.949	1.425	−1.023	−1.92	−0.126	−2.337	28	0.027

* For difference between groups in change over time.

**Table 6:** Comparison of Change in Salivary Viral Load from Baseline to Day 60 between Control and Test AFTER Adjusting for Baseline Value.

		Control						Test				
		Day 0		Day 60			Day 0		Day 60		Mean Difference	F-test*
	N	Adjusted Mean	SE	Adjusted Mean	SE	N	Adjusted Mean	SE	Adjusted Mean	SE		p-value
**HSV- 1**	15	5.044	0.0 00	3.205	0.114	15	5.044	0.000	0.963	0.114	−2.242	p<0.001
**CMV**	15	6.336	0.0 00	4.421	0.209	15	6.336	0.000	0.379	0.209	−4.042	p<0.001
**EBV**	15	6.985	0.0 00	3.657	0.145	15	6.985	0.000	1.437	0.145	−2.220	p<0.001

* For difference between groups in change over time.
